# Editorial: Psychometrics in psychiatry 2022: mood disorders

**DOI:** 10.3389/fpsyt.2024.1413839

**Published:** 2024-04-29

**Authors:** Daniel Stjepanović

**Affiliations:** ^1^ National Centre For Youth Substance Use Research, Faculty of Health and Behavioural Sciences, The University of Queensland, St Lucia, QLD, Australia; ^2^ School of Psychology, Faculty of Health and Behavioural Sciences, The University of Queensland, St Lucia, QLD, Australia

**Keywords:** psychiatry, psychometrics, mood disorder, anxiety, depression

The scientific revolution has from its earliest moments focused on measurement, with the early focus seeking to identify scales that could reliably and consistently measure physical properties such as temperature, pressure and so on. This need to measure and define was reflected in psychology and psychiatry, resulting in the field of psychometrics. Francis Galton (1) provided one of the earliest descriptions of psychometrics as the “art of imposing measurement and number upon operations of the mind”. While this definition is still in spirit accurate, the field of psychometrics has moved from being an art to a science, relying on rigorous methods and sophisticated statistical tools to “impose measurement” upon the mind. This Research Topic brings together seven papers which advance psychometric tools or apply existing ones to understanding psychiatric phenomena.

Four papers translated or examined the psychometric properties of existing scales in local populations. The first, by Baig et al. translated the Children’s Emotion Management Scale (CEMS) to Urdu and estimated the reliability and validity of their translated version in a Pakistani sample. The CEMS provides a measure of emotion regulation which are cognitive strategies used to monitor and modify emotional reactions. The need for a localised version of the CEMS in Urdu is highlighted by the high prevalence of emotional and behavioural problems amongst young people in Pakistan. The authors examined cross-language validation, internal consistency and split-half reliability, and convergent and divergent validity in a number of distinct samples. Results indicated sound psychometric properties, providing Urdu speakers with a reliable and valid translation of the CEMS.

The second paper by Le-Nguyen-Thuy et al. provides a forward-backward translation into Vietnamese of the 17-item Hamilton Depression Rating Scale (Hamilton D-17). The Hamilton D-17 provides an important tool in assessing and guiding the evaluation of recovery from depression. The lack of a validated Vietnamese version hampers the ability of clinicians to routinely use the Hamilton D-17 in diagnosis and treatment. Following translation, the authors recruited a sample of patients from University Medical Center of Medicine and Pharmacy University at Ho Chi Minh City. The Vietnamese translation of the Hamilton D-17 demonstrated high levels of validity and reliability, enabling its routine use in Vietnam.

Mehrabi et al. translated the Suicide Ideation and Behavior Scale (SIBS) into Farsi in the third paper in this Research Topic. Reliability and validity were then examined in a highly burdened Afghan student sample. Confirmatory factor analysis returned a single factor, consistent with others’ work using the SIBS. The authors also demonstrated construct validity of their scale against the SBQ-R, a measure of depression, PTSD and suicide ideation. Given the unfortunately high rates of suicidal ideation and behaviour in Afghanistan, the authors’ contribution will provide a useful tool to alert clinicians to instances of suicidal ideation or behaviour that require further examination.

And lastly, Ma et al. examined the psychometric properties of the Affect Lability Scale-Short Form (ALS-SF) into Chinese. Affective lability is the tendency for emotional states to change rapidly, unpredictably and excessively, and may be a contributor or feature of depression and bipolar disorder. While the ALS-SF and its psychometric properties have been established in adolescents in China, this is the first study to expand this to adults.

The final three papers focus on the application of existing scales to understand psychiatric symptoms. Ihler et al. sought to elucidate the latent structure of negative symptoms experienced in bipolar disorder. To do this, they recruited a large sample of individuals with bipolar disorder and explored the factor structure of negative symptoms as measured with the Positive and Negative Syndrome Scale (PANSS). Results indicated a two factor structure underlying negative symptoms in bipolar disorder: diminished expression and apathy. This mirrors the structure observed in non-affective psychotic disorders, indicating a potential link with bipolar disorder. This suggests utility of using negative symptoms as a transdiagnostic phenomenon, as has been suggested in previous research.

Bartosik et al. sought to understand the association of affective temperament and depression in a sample of Polish medical students. This is an important work given the high levels of stress and elevated rates of depression experienced by medical students. The results showed a positive correlation between almost all affective temperament types and the severity of depressive symptoms as measured using the Beck Depression Inventory. The strongest association, however, was between anxious temperament and depressive symptoms, potentially reflecting the strong association of the two disorders. Future work is necessary to expand this knowledge beyond this sample to understand if these results translate beyond medical students.

Lastly, Yang et al.’s study utilised a distinctly novel approach. The authors measured the levels of peripheral immune cells and psychiatric symptoms. In a case control study, a group of newly diagnosed patients was compared to matched controls, noting higher immune cell levels in peripheral blood in those with psychiatric symptoms relative to controls, although some markers only differentiated those with multiple psychiatric symptoms against controls. Additionally, sleep quality was noted to be worse in those with psychiatric symptoms. The results are intriguing and suggest the need to further investigate the relationships between immune function, psychiatric symptoms and sleep.

In conclusion, the works that form this Research Topic will further psychometric research and the use of psychometrics in psychiatry. Expanding the use of existing scales to new linguistic and cultural communities will enable better diagnosis, treatment and cross-cultural comparison. Novel examinations of psychometric tools to understand psychiatric phenomenology, such as the conceptualisation of negative symptoms as a transdiagnostic feature, could spur the better understanding of psychiatric disease and spur the development of more precise, valid and reliable psychometric tools. Thank you to the reviewers for their insightful and constructive feedback and I hope that this Research Topic will inspire future work.

## Author contributions

DS: Writing – original draft, Writing – review & editing.
